# Respiratory-Support Phenotypes and In-Hospital Mortality in Pediatric Postprocedural Respiratory Complications

**DOI:** 10.3390/healthcare14172805

**Published:** 2026-09-01

**Authors:** Michael Samawi, Hani Samawi, Gulzar H. Shah, Majd Al-Saleh

**Affiliations:** 1 Defense Language Institute Foreign Language Center, Monterey, CA 93944, USA; 2 Jiann-Ping Hsu College of Public Health, Georgia Southern University, P.O. Box 8015, Statesboro, GA 30460, USA; hsamawi@georgiasouthern.edu (H.S.); gshah@georgiasouthern.edu (G.H.S.); ma22524@georgiasouthern.edu (M.A.-S.)

**Keywords:** postoperative respiratory complications, pediatric surgery, mechanical ventilation, ECMO, mortality, HCUP KID, respiratory-support phenotypes, administrative data phenotyping

## Abstract

**Highlights:**

**What are the main findings?**

- Respiratory-support phenotypes were strongly associated with in-hospital mortality, with survey-weighted mortality ranging from 0.73% without coded MV/intubation/ECMO to 35.46% with ECMO support.
- Mortality was not monotonic with ventilation duration; in sensitivity analysis, qualifying postoperative intubation showed a stronger association with mortality than 24–96 h mechanical ventilation alone.

**What are the implications of the main findings?**

- Hospital-course respiratory support phenotypes may provide more clinically informative postoperative surveillance than a single binary respiratory-complication measure.
- These phenotypes should be viewed as hypothesis-generating tools for quality review and require external clinical validation before use for bedside risk stratification or comparative performance assessment.

**Abstract:**

**Background/Objectives:** Postprocedural respiratory complications encompass heterogeneous patterns of invasive respiratory support. We examined whether hospital-course respiratory-support phenotypes were associated with in-hospital mortality among operative pediatric discharges. **Methods:** We analyzed the 2022 Healthcare Cost and Utilization Project Kids’ Inpatient Database. Discharges with Clinical Classifications Software Refined category RSP017 were restricted to those with a major operating-room procedure. AHRQ Pediatric Quality Indicator 09 timing criteria anchored postoperative mechanical ventilation and intubation. Survey-weighted logistic regression accounted for KID strata, hospital clustering, and discharge weights, and adjusted for age, sex, multisystem complication involvement, AHRQ nonweighted comorbidity burden, and procedure domain. **Results:** Of 7378 operative RSP017 discharges, 6358 were aged ≤17 years and 6334 entered the final model, with 216 deaths. Survey-weighted mortality was 35.46% with ECMO support, 7.35% with postoperative 24–96 h ventilation/intubation, 4.71% with postoperative ventilation > 96 h, 4.11% with other invasive support, and 0.73% in the reference phenotype. Corresponding adjusted odds ratios were 60.12, 8.76, 5.28, and 4.85. Before hierarchical assignment, 350/6358 discharges (5.50%) met more than one candidate phenotype definition. Alternative hierarchy and operative-anchor analyses yielded similar associations. When the combined phenotype was disaggregated, qualifying postoperative intubation had a stronger association with mortality than 24–96 h ventilation alone. **Conclusions:** Administrative respiratory-support phenotypes were associated with markedly different in-hospital mortality. Findings were robust to alternative classification and timing rules, but the framework is hypothesis-generating and requires external clinical validation.

## 1. Introduction

Postprocedural respiratory complications remain among the most consequential adverse events in pediatric surgical care. Mechanical ventilation, postoperative intubation, prolonged respiratory support, and extracorporeal membrane oxygenation (ECMO) may occur in the same broad administrative complication category, yet they represent different hospital-course patterns and may be associated with substantially different outcomes [1,2].

The Agency for Healthcare Research and Quality (AHRQ)’s Pediatric Quality Indicator 09 (PDI-09) provides a standardized framework for surveillance of postoperative respiratory failure among eligible pediatric surgical discharges. Its timing logic distinguishes prolonged mechanical ventilation and postoperative intubation relative to the first major operating-room (OR) procedure [3]. However, PDI-09 is an occurrence-based quality indicator and does not by itself describe heterogeneity in respiratory-support patterns after a postprocedural respiratory complication has been coded.

Prior national work has identified demographic, clinical, procedural, and hospital factors associated with pediatric postoperative respiratory failure [1,2]. The contemporary pediatric ventilation and ECMO literature likewise emphasizes that respiratory support is clinically heterogeneous and that duration, escalation, and liberation occur within different physiologic contexts [4,5,6,7,8]. National administrative data cannot reproduce continuous bedside respiratory courses, but procedure codes and procedure-day information can support reproducible hospital-course respiratory-support phenotypes.

To examine this heterogeneity, we analyzed the 2022 HCUP Kids’ Inpatient Database (KID). We identified operative discharges with the official HCUP Clinical Classifications Software Refined (CCSR) category RSP017 for postprocedural or postoperative respiratory system complication, anchored postoperative respiratory-support timing to major OR procedures, and classified mutually exclusive respiratory-support phenotypes. The primary outcome was directly observed in-hospital mortality.

We hypothesized that hospital-course respiratory-support phenotypes would be associated with different odds of in-hospital mortality after adjustment for age, sex, comorbidity burden, multisystem postoperative complication involvement, and procedure domain. The analysis was designed as an observational association study rather than a clinical prediction model or causal treatment-effect analysis.

## 2. Materials and Methods

### 2.1. Study Design and Data Source

We conducted a retrospective cross-sectional analysis of discharge-level data from the 2022 HCUP KID, sponsored by AHRQ. The KID is the largest publicly available all-payer pediatric inpatient database in the United States and uses a complex sampling design that supports national discharge-level inference when the supplied survey weights, strata, and hospital clusters are incorporated [9]. The 2022 KID contains 3,009,812 sampled discharges. All reported sample counts are unweighted; regression estimates account for the KID design.

The study period was the calendar year 2022. The unit of analysis was an inpatient discharge. Statistical analyses were performed in SAS 9.4 with SAS/STAT (SAS Institute Inc., Cary, NC, USA).

### 2.2. Cohort Identification and Operative Anchor

We identified discharges assigned to CCSR diagnosis category RSP017, postprocedural or postoperative respiratory system complication. CCSR is an AHRQ/HCUP taxonomy that aggregates ICD-10-CM diagnosis codes into clinically coherent categories [10]. We then restricted the cohort to discharges with at least one major OR procedure identified with HCUP Procedure Classes Refined classes 3 or 4 [11]. This broader operative RSP017 cohort was used to characterize respiratory-support phenotypes and does not claim to reproduce the full PDI-09 denominator. Operational cohort and respiratory-support definitions are provided in Appendix A.1 (Table A1).

For each discharge, the primary operative anchor was the first hospital day containing a dated major procedure (Procedure Classes Refined class 3 or 4). If multiple qualifying major procedures occurred on the same hospital day, that shared day served as the anchor because KID does not provide within-day procedure sequencing. If major procedures occurred on more than one hospital day, the earliest dated major procedure was the primary anchor, consistent with the first-major-OR timing framework used by PDI-09 [3,11]. To assess whether a later operation could materially alter phenotype assignment, we repeated the timing classification using the last dated major OR as a sensitivity analysis.

**Figure 1:**
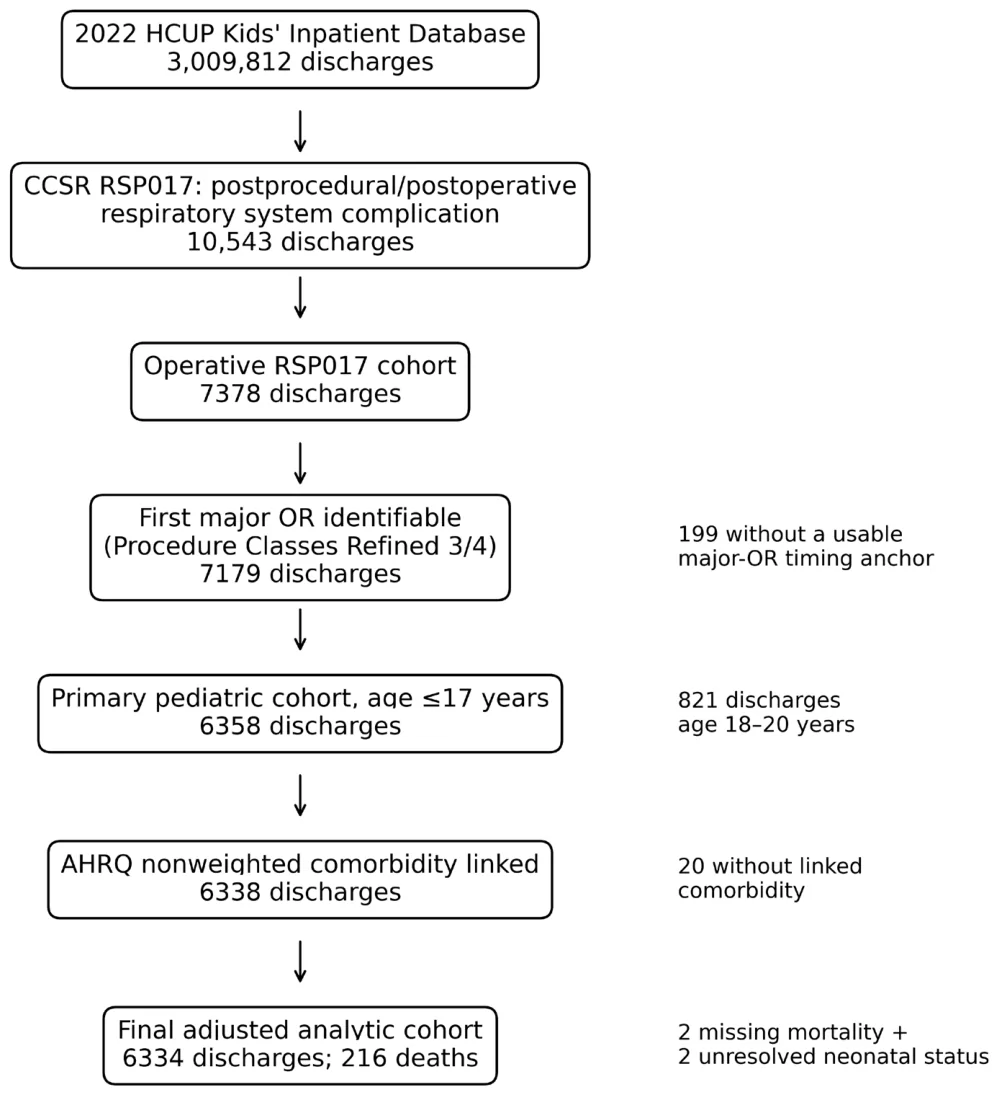
Cohort derivation for the primary mortality analysis. Counts are unweighted KID sample discharges. RSP017 denotes the CCSR category for postprocedural or postoperative respiratory system complication.

Because the 2022 KID does not provide day-level age (AGEDAY), neonatal status among AGE = 0 discharges was classified with the HCUP AGE_NEONATE indicator [9]. Two age-zero discharges had missing AGE_NEONATE and therefore could not be assigned to the prespecified neonatal or infant stratum. Exclusions from the final adjusted model were limited to records without a usable age classification, missing in-hospital mortality, or unavailable linked AHRQ nonweighted comorbidity burden. Figure 1 and Table A2 summarize cohort construction.

### 2.3. AHRQ-Anchored Respiratory-Support Phenotypes

AHRQ PDI-09 criteria were used to anchor the timing of postoperative respiratory support but were not used to claim reproduction of PDI-09 itself. Under version 2024 specifications, qualifying timing includes mechanical ventilation > 96 h with the last dated occurrence on or after the first major OR; mechanical ventilation 24–96 h with the last dated occurrence at least 2 days after the first major OR; and qualifying postoperative intubation with the last dated occurrence at least 1 day after the first major OR [3]. Exact ICD-10-PCS timing codes and the timing-anchor quality audit are documented in Appendix A.3 (Table A3).

Five mutually exclusive hospital-course phenotypes were defined hierarchically: (1) ECMO support during the hospitalization; (2) postoperative mechanical ventilation > 96 h meeting the timing rule; (3) postoperative mechanical ventilation 24–96 h or qualifying postoperative intubation; (4) other invasive mechanical ventilation/intubation support that did not meet the preceding timed definitions; and (5) no coded mechanical ventilation, intubation, or ECMO. ECMO was assigned first to isolate hospitalizations involving extracorporeal support regardless of concurrent ventilation. Among non-ECMO discharges, >96 h ventilation was assigned before the 24–96 h/intubation category to resolve overlapping respiratory-support codes into mutually exclusive categories. The hierarchy was not intended as an ordinal severity scale.

Before hierarchical assignment, we quantified how many discharges met more than one candidate phenotype definition and documented the overlap patterns and resulting assignment. The primary hierarchy was fixed before the mortality models and was not altered in response to outcome cell counts. Sensitivity analyses (i) prioritized >96 h ventilation before ECMO, (ii) prioritized the 24–96 h/intubation category before >96 h ventilation while retaining ECMO first, and (iii) separated 24–96 h ventilation from qualifying postoperative intubation. These analyses tested whether the principal findings depended on a single classification rule.

### 2.4. Multisystem Complication Involvement, Comorbidity, and Procedure Domain

Multisystem involvement was defined as the respiratory complication plus at least one additional postoperative complication system identified in the analytic phenotype file. This is an administrative complication construct rather than a validated physiologic organ-dysfunction score and is used only as a marker of broader coded postoperative complication involvement [12,13,14].

Comorbidity burden was obtained from a previously generated 2022 AHRQ Quality Indicators analytic lineage and linked back to current KID discharges by discharge and hospital identifiers. The source was deduplicated to one record per discharge before linkage. Comorbidity values were internally consistent across duplicate source rows; 6338 of 6358 primary-cohort records linked successfully (99.69%), and immutable discharge characteristics showed complete agreement in the validation audit (Appendix A, Table A4). Only the AHRQ nonweighted comorbidity count and its PDI-09 category indicators were imported; mortality and age-in-days variables from the historical analytic lineage were not used. The recovered indicators conformed exactly to the AHRQ PDI-09 count categories, with category 1 representing 1–2 counted conditions and category 2 representing ≥3 conditions [15,16].

Procedure domain was represented by six mutually exclusive categories derived from HCUP procedural CCSR clinical domains: cardiovascular; neurologic/ear, nose, and throat; musculoskeletal; gastrointestinal/hepatobiliary; respiratory; and other principal procedures [17]. This study-specific collapse was used for case-mix adjustment and should not be interpreted as a validated procedure-severity index.

### 2.5. Outcome

The primary outcome was in-hospital mortality, defined from the 2022 KID DIED indicator. Mortality was selected as a directly observed, clinically unambiguous endpoint. Two records in the age-eligible cohort had missing mortality status and were excluded from mortality models.

### 2.6. Statistical Analysis

Descriptive analyses report unweighted sampled-discharge counts and proportions unless explicitly labeled as survey-weighted. To assess potential selection from the 24 age-eligible records excluded from the final adjusted model, we descriptively compared included and excluded records on age, sex, phenotype, multisystem involvement, procedure domain, observed mortality when available, and length of stay. Because the excluded group was small, no null-hypothesis significance tests were used for this comparison. Survey-weighted absolute mortality by phenotype and 95% confidence intervals were estimated with PROC SURVEYMEANS. Crude absolute mortality differences versus the reference phenotype were estimated with PROC SURVEYREG.

Crude phenotype associations and multivariable mortality models used PROC SURVEYLOGISTIC with KID_STRATUM as the stratum variable, HOSP_KID as the cluster variable, and DISCWT as the discharge weight. Taylor-series linearization was used for variance estimation. The full 2022 KID design file was retained, and the response was set missing outside the analytic subpopulation with NOMCAR rather than physically subsetting the survey design. The full KID contained 95 strata and 3811 hospital clusters; no values of DISCWT, KID_STRATUM, or HOSP_KID were missing in either the full KID or final analytic cohort. Although the final cohort represented 51 strata and 400 hospitals, all 95 strata and 3811 clusters were retained for variance estimation.

The prespecified primary model included respiratory-support phenotype, multisystem involvement, pediatric age category, sex, AHRQ nonweighted comorbidity burden, and procedure domain. Age was categorized as neonate (first 28 days), infant 29 days to <1 year, 1–4 years, 5–9 years, 10–14 years, and 15–17 years; age 1–4 years was the reference. Comorbidity burden was categorized as 0, 1–2, or ≥3 conditions, consistent with the AHRQ nonweighted PDI-09 comorbidity construct [15,16]. Adjusted odds ratios (aORs), 95% confidence intervals (CIs), and two-sided *p*-values are reported. Odds ratios are interpreted on the odds scale and not as risk ratios.

Sensitivity analyses removed procedure domain from the covariate set, excluded ECMO occurring before the first major OR or with unknown timing, compared identical no-comorbidity models in the full eligible and comorbidity-complete samples, applied two alternative phenotype hierarchies, separated 24–96 h ventilation from qualifying postoperative intubation, and rebuilt timed phenotypes using the last rather than first major OR. The study was not designed to develop or compare clinical prediction models; therefore, calibration, reclassification, and decision-curve analyses were not performed. Model fit statistics and the c-statistic are retained only in Appendix A as reproducibility diagnostics and are not interpreted as evidence of predictive validity. All statistical analyses were performed using SAS 9.4 with SAS/STAT (SAS Institute Inc., Cary, NC, USA).

## 3. Results

### 3.1. Cohort Derivation and Analytic Sample

Among 3,009,812 sampled KID discharges, 10,543 had RSP017. The operative RSP017 cohort included 7378 discharges, of which 7179 (97.30%) had an identifiable dated major OR. The primary age ≤ 17 cohort comprised 6358 discharges. Of these, 20 lacked linked AHRQ nonweighted comorbidity information; two had missing in-hospital mortality, and two age-zero discharges had unresolved AGE_NEONATE status. These mutually exclusive exclusions yielded a final adjusted cohort of 6334 discharges with 216 deaths (Figure 1).

The 24 excluded records represented 0.38% of the age-eligible cohort. Descriptively, excluded records had longer hospitalizations than included records (median length of stay was 38 days [IQR 13–91] among 23 with nonmissing LOS versus 12 days [IQR 4–37] among 6279 included records with nonmissing LOS) and a somewhat higher proportion with multisystem involvement (29.17% versus 20.73%). Female sex was similar (45.83% versus 41.77%). Among excluded records, two had ECMO support; three had postoperative ventilation > 96 h; 12 had other invasive support, and seven were in the reference phenotype; one death was observed among 22 records with known mortality. Full descriptive comparisons are provided in Table A5. The previously prespecified no-comorbidity selection sensitivity yielded nearly identical phenotype estimates in the all-eligible and comorbidity-complete samples. Characteristics of the final adjusted analytic cohort are summarized in Table 1.

### 3.2. Respiratory-Support Phenotype Assignment and Absolute Mortality

Before the hierarchy was applied, 350 of 6358 age-eligible discharges (5.50%) met at least two candidate phenotype definitions: 328 met two candidate categories and 22 met three. The most common overlap was postoperative MV > 96 h plus the 24–96 h/intubation category (*n* = 215). Among ECMO-positive overlaps, 56 also met >96 h ventilation; 45 also met other invasive support; 22 met both >96 h and 24–96 h/intubation, and 12 also met 24–96 h/intubation. Under the frozen hierarchy, all 135 ECMO-overlap discharges were assigned to ECMO and the 215 non-ECMO discharges meeting both timed ventilation categories were assigned to >96 h ventilation (Table A6).

In the final analytic cohort, 205 discharges were classified as ECMO support, 846 as postoperative mechanical ventilation > 96 h, 227 as postoperative mechanical ventilation 24–96 h or qualifying intubation, 1433 as other/short/non-postoperatively timed invasive support, and 3623 as no coded MV/intubation/ECMO. Survey-weighted mortality was 35.46% (95% CI 28.21–42.71), 4.71% (3.22–6.20), 7.35% (4.00–10.71), 4.11% (3.04–5.18), and 0.73% (0.42–1.04), respectively. Relative to the reference, crude survey-weighted absolute mortality differences were +34.73, +3.98, +6.62, and +3.38 percentage points, respectively (Table 2).

### 3.3. Primary Survey-Weighted Mortality Model

**Figure 2:**
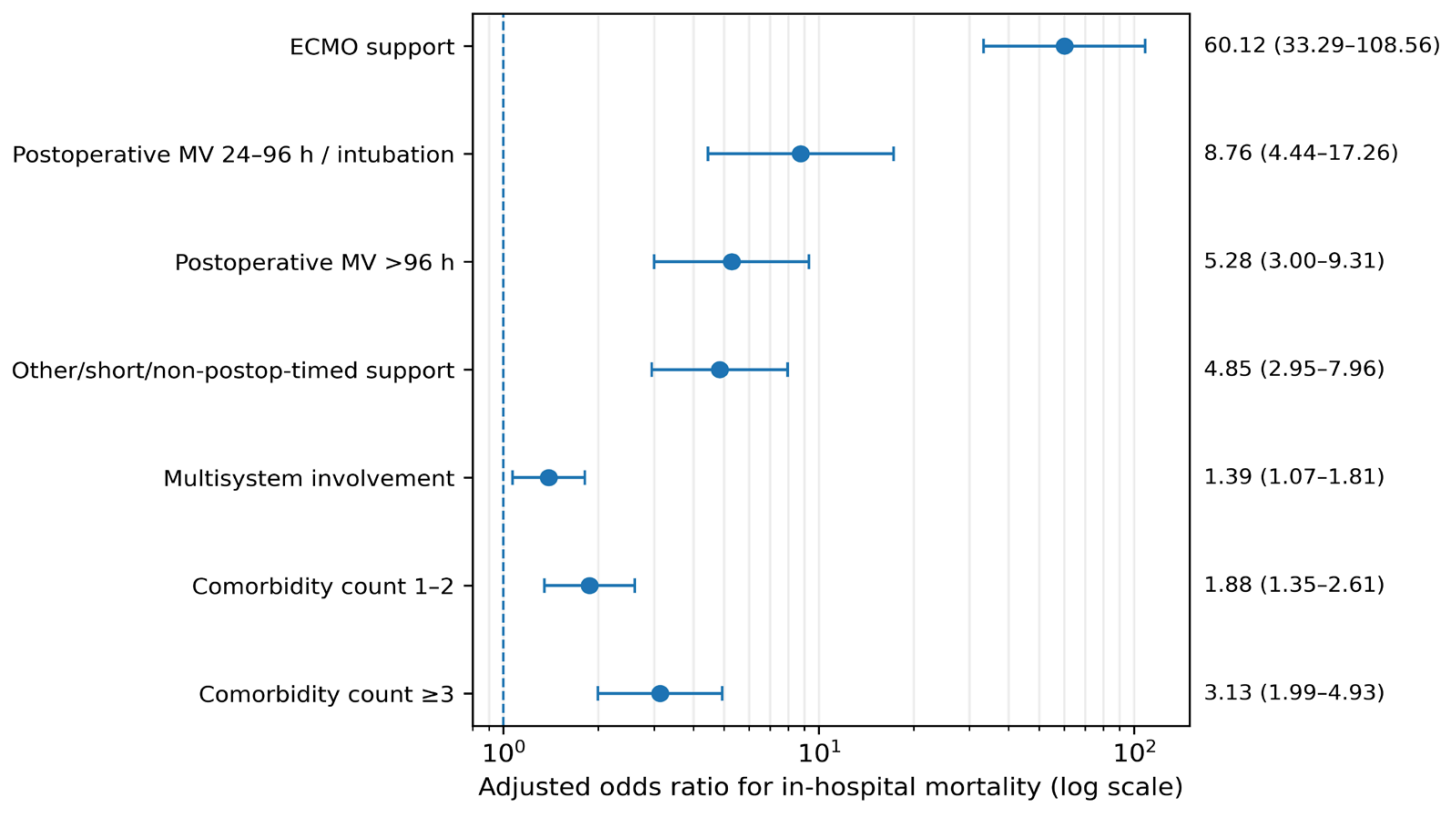
Selected adjusted associations with in-hospital mortality from the primary survey-weighted model. Points represent adjusted odds ratios and horizontal bars represent the 95% confidence intervals. The x-axis is logarithmic. Odds ratios describe associations on the odds scale and are not risk ratios.

The primary survey-weighted model used all 6334 complete analytic records and converged normally. Respiratory-support phenotype was associated with mortality overall (Type III, *p* < 0.0001). Relative to the no-coded-MV/intubation/ECMO reference phenotype, adjusted mortality odds were 60.12 (95% CI 33.29–108.56) for ECMO support, 5.28 (3.00–9.31) for postoperative MV > 96 h, 8.76 (4.44–17.26) for postoperative MV 24–96 h/intubation, and 4.85 (2.95–7.97) for other invasive support (Table 3; Figure 2).

Multisystem complication involvement was associated with higher mortality odds in the primary model (aOR 1.39, 95% CI 1.07–1.82; *p* = 0.0139). Comorbidity burden demonstrated a graded association: 1–2 conditions versus zero, aOR of 1.88 (1.35–2.61), and ≥3 versus zero, aOR of 3.13 (1.99–4.93), both *p* < 0.0001. Sex was not associated with mortality after adjustment (aOR of 1.10, 95% CI 0.80–1.51; *p* = 0.5650).

Procedure domain was associated with mortality overall (*p* = 0.0060). Relative to cardiovascular procedures, neurologic/ENT procedures had an aOR of 2.34 (95% CI 1.43–3.85), gastrointestinal/hepatobiliary procedures of 1.74 (1.06–2.85), and respiratory procedures of 1.67 (1.03–2.71). The complete coefficient set is provided in Table A7.

### 3.4. Sensitivity Analyses

Previously specified sensitivity analyses were stable. Removing procedure domain produced phenotype aORs of 59.00 for ECMO, 5.75 for postoperative >96 h ventilation, 9.09 for postoperative 24–96 h/intubation, and 5.29 for other invasive support. Excluding ECMO before the first major OR or with unknown timing yielded corresponding aORs of 60.74, 5.22, 8.65, and 4.81 among 6283 discharges with 200 deaths. The no-comorbidity models were nearly identical in all eligible records and the comorbidity-complete subset (Table 4 and Table A8).

The phenotype findings were also robust to alternative hierarchy rules. When postoperative MV > 96 h was prioritized before ECMO, aORs were 51.72 for ECMO, 7.99 for >96 h ventilation, 8.55 for 24–96 h/intubation, and 4.74 for other invasive support. When the 24–96 h/intubation category was prioritized before >96 h ventilation while ECMO remained first, the corresponding aORs were 60.46, 4.20, 8.82, and 4.86. Thus, the main associations did not depend on a single hierarchy ordering (Table A8).

A six-level sensitivity separated the components of the combined postoperative 24–96 h/intubation phenotype. Qualifying postoperative intubation showed a strong association with mortality (aOR of 13.76, 95% CI 6.92–27.36), whereas postoperative MV 24–96 h alone had a smaller and imprecise estimate (aOR of 2.38, 95% CI 0.61–9.34). In the same model, ECMO, >96 h ventilation, and other invasive support had aORs of 60.90, 5.30, and 4.87, respectively (Table A9).

### 3.5. Operative-Anchor and Additional Course Sensitivity Analyses

Among final-cohort records, 2115 (33.39%) had one dated major procedure; 2619 (41.35%) had multiple major procedures on the same hospital day, and 1600 (25.26%) had major procedures spanning more than one day. Rebuilding timed phenotypes from the last rather than first major OR reclassified 502 of 6334 discharges (7.93%), but adjusted associations remained similar: ECMO, aOR of 59.49; postoperative >96 h ventilation, 6.25; postoperative 24–96 h/intubation, 9.26; other invasive support, 4.87; and multisystem involvement, 1.41 (Table A8 and Table A10).

Phenotype 4 remained heterogeneous. Among 1433 final-cohort discharges in this category, mortality was 8.64% (49/567) when a >96 h ventilation code was present but did not satisfy postoperative timing and 1.15% (10/866) otherwise. Postoperative tracheostomy was examined descriptively as a prolonged-course marker but was not included in the primary hierarchy because its timing is particularly vulnerable to survivor conditioning and cannot be interpreted as an early postoperative exposure (Table A11).

## 4. Discussion

### 4.1. Principal Findings

In this survey-weighted national analysis of pediatric operative discharges with postprocedural respiratory complications, hospital-course respiratory-support phenotypes were associated with markedly different in-hospital mortality. The ECMO phenotype had the highest absolute mortality and the largest adjusted odds ratio, while the other invasive-support categories also differed substantially from the reference group. These associations remained similar across alternative phenotype hierarchies, ECMO-timing restrictions, procedure-domain specifications, missing-comorbidity analyses, and a last-major-OR timing anchor.

A second important finding is that the primary phenotype categories should not be read as an ordinal severity scale. The combined postoperative 24–96 h ventilation/intubation category had higher mortality than the >96 h ventilation category in the primary analysis, but the disaggregated sensitivity showed that this pattern was driven principally by qualifying postoperative intubation rather than 24–96 h ventilation alone. This distinction sharpens the interpretation of the primary model and argues against equating longer coded ventilation duration with uniformly higher mortality.

### 4.2. Interpreting the Non-Monotonic Phenotype Pattern

Mechanical ventilation duration is partly conditioned on survival, physiology, operative timing, escalation decisions, and successful liberation. A patient who dies or undergoes abrupt escalation may not accumulate prolonged ventilation duration, whereas a child who survives a difficult postoperative course may remain ventilated for many days. Administrative timing categories therefore capture different hospital-course states rather than successive steps on a single severity continuum.

The disaggregated sensitivity analysis supports this interpretation. Qualifying postoperative intubation was associated with markedly higher mortality odds, whereas the estimate for 24–96 h mechanical ventilation alone was smaller and imprecise. The KID cannot determine whether qualifying intubation followed extubation failure, airway compromise, planned reintubation, progressive pulmonary disease, or another indication. Accordingly, these categories should be viewed as reproducible administrative phenotypes that identify groups for further clinical investigation, not as mechanistic explanations or treatment effects.

### 4.3. ECMO as a Hospital-Course Support Phenotype

ECMO support was associated with approximately 60-fold higher adjusted odds of mortality relative to the no-coded-MV/intubation/ECMO reference phenotype, and survey-weighted mortality was 35.46%. This estimate reflects the extreme underlying acuity of ECMO-treated hospitalizations as well as the support modality itself and must not be interpreted as a causal effect of ECMO. Contemporary pediatric ECMO studies similarly describe populations with substantial illness burden and mortality [7,8].

Excluding ECMO that occurred before the first major OR or had unknown timing left the adjusted estimate essentially unchanged. Reordering the hierarchy so that >96 h postoperative ventilation took precedence over ECMO also left ECMO strongly associated with mortality. These analyses reduce concern that the main ECMO association is simply an artifact of temporal anchoring or hierarchy priority, while still leaving clinical indication and disease severity unresolved.

### 4.4. Multisystem Involvement and Comorbidity Burden

Multisystem postoperative complication involvement was associated with mortality in the primary model, but the association was modest and somewhat sensitive to case-mix specification: when procedure domain was omitted, the estimate attenuated to 1.27 (95% CI 0.98–1.65). This pattern supports cautious interpretation of multisystem involvement as an administrative marker of broader postoperative complication burden rather than an independent physiologic organ-failure construct.

AHRQ nonweighted comorbidity burden showed a clearer graded association with mortality. Compared with children without counted AHRQ comorbidities, adjusted mortality odds were approximately 1.9-fold higher with 1–2 conditions and 3.1-fold higher with ≥3. This direction is consistent with the broader literature on pediatric medical complexity, including the 2024 update to the complex chronic conditions framework [18]. Importantly, the respiratory-support phenotype associations remained substantial after adjustment for this baseline vulnerability.

### 4.5. Clinical and Quality-Improvement Implications

These findings are most appropriately used for hypothesis generation, administrative surveillance, and structured quality review rather than bedside treatment selection or comparative performance ranking. An occurrence-based indicator establishes that a postoperative respiratory complication was coded. The phenotype framework adds information about coded respiratory support and timing within the hospitalization, which may help identify distinct groups for targeted chart review.

The KID cannot establish whether an individual phenotype reflects a preventable complication, appropriate escalation, underlying disease severity, planned postoperative management, or institutional practice. A qualifying postoperative intubation, prolonged postoperative ventilation, and ECMO support therefore should not automatically be interpreted as equivalent quality failures or treatment pathways. External validation using clinical data with ventilator settings, indications, extubation events, and continuous timing is required before these phenotypes are used for bedside stratification or interhospital quality comparisons.

### 4.6. Strengths and Limitations

This study has several strengths. Cohort identification uses an official CCSR diagnosis category with an operative restriction based on Procedure Classes Refined; respiratory-support timing is anchored to major OR procedures, and the KID complex survey design is incorporated through discharge weights, strata, and hospital clusters. The analysis retained nearly all age-eligible records, quantified the characteristics of the small excluded group, reported survey-weighted absolute mortality and absolute mortality differences, audited pre-hierarchy overlap, and tested alternative hierarchy and operative-anchor definitions. The raw-code reconstruction reproduced the frozen primary phenotype assignments for all 6358 age-eligible records.

Limitations should constrain interpretation. Administrative data do not provide ventilator settings, positive end-expiratory pressure, arterial blood gases, oxygenation indices, PRISM scores, continuous bedside timing, extubation status, or the clinical indication for intubation. ICD-10-CM/PCS codes may be incomplete or misclassified. The RSP017 cohort is broader than PDI-09, and this study does not reproduce the AHRQ indicator. KID procedure days cannot establish within-day sequencing; when multiple major procedures occur on the same day, a common day anchor is unavoidable. When procedures span multiple days, the first major OR may not always be the procedure most clinically related to later respiratory support, although the last-major-OR sensitivity produced similar mortality associations.

The phenotypes are hospital-course administrative categories, not continuous clinical courses, and some respiratory-support components may occur after substantial deterioration has already developed. Residual confounding by illness severity is likely. Adjusted odds ratios should not be interpreted causally or as risk ratios. ECMO and postoperative intubation may reflect appropriate escalation rather than preventable harm. The multisystem variable is an administrative complication construct rather than a validated physiologic organ-dysfunction score. Phenotype 4 is heterogeneous; tracheostomy is vulnerable to survivor conditioning, and the analysis covers one KID release year. External validation in other years and clinical datasets is required. Additional reproducibility details are provided in Appendix A.11.

## 5. Conclusions

Among pediatric operative discharges with postprocedural respiratory complications, hospital-course respiratory-support phenotypes were associated with markedly different in-hospital mortality after adjustment for measured patient and procedural factors. The findings were robust to alternative phenotype hierarchies and to use of the last rather than first major OR as the timing anchor. Disaggregation of the combined 24–96 h ventilation/intubation category showed that qualifying postoperative intubation, rather than 24–96 h ventilation alone, accounted for the stronger mortality association in that category. These administrative phenotypes are hypothesis-generating tools for surveillance and structured review and require external clinical validation before bedside or comparative-quality use.

## Figures and Tables

**Table 1:** Characteristics of the final adjusted analytic cohort (*n* = 6334).

Characteristic	*n*	%
Respiratory-support phenotype		
ECMO support	205	3.24
Postoperative MV > 96 h	846	13.36
Postoperative MV 24–96 h or intubation	227	3.58
Other/short/non-postop-timed invasive support	1433	22.62
No coded MV/intubation/ECMO	3623	57.20
Multisystem involvement	1313	20.73
Age		
Neonate, first 28 days	1150	18.16
Infant, 29 d- < 1 y	1448	22.86
1–4 y	1284	20.27
5–9 y	798	12.60
10–14 y	872	13.77
15–17 y	782	12.35
Sex		
Male	3688	58.23
Female	2646	41.77
AHRQ nonweighted comorbidity burden		
0	2740	43.26
1–2	2995	47.28
≥3	599	9.46
Procedure domain		
Cardiovascular	2352	37.13
Neurologic/ENT	757	11.95
Musculoskeletal	616	9.73
GI/hepatobiliary	557	8.79
Respiratory procedure	966	15.25
Other principal procedure	1086	17.15
In-hospital death	216	3.41

Note: Percentages use the final adjusted model denominator. ENT, ear, nose, and throat; GI, gastrointestinal; ECMO, extracorporeal membrane oxygenation; MV, mechanical ventilation.

**Table 2:** Respiratory-support phenotype, observed and survey-weighted mortality, absolute mortality difference, and adjusted association with in-hospital death.

Phenotype	*n*	Deaths, *n* (%)	Weighted Mortality, % (95% CI)	Difference vs. Reference, Percentage Points (95% CI)	Adjusted OR (95% CI)
ECMO support	205	73 (35.61)	35.46 (28.21–42.71)	+34.73 (27.50–41.95)	60.12 (33.29–108.56)
Postoperative MV > 96 h	846	40 (4.73)	4.71 (3.22–6.20)	+3.98 (2.44–5.51)	5.28 (3.00–9.31)
Postoperative MV 24–96 h/intubation	227	17 (7.49)	7.35 (4.00–10.71)	+6.62 (3.26–9.98)	8.76 (4.44–17.26)
Other/short/non-postop-timed support	1433	59 (4.12)	4.11 (3.04–5.18)	+3.38 (2.29–4.46)	4.85 (2.95–7.97)
No coded MV/intubation/ECMO	3623	27 (0.75)	0.73 (0.42–1.04)	Reference	Reference

Note: *n* and deaths are unweighted sampled discharges. Survey-weighted mortality and absolute mortality differences use DISCWT with the full KID strata and cluster design. Absolute differences are percentage-point differences versus the no-coded-MV/intubation/ECMO reference. Adjusted odds ratios are from the primary survey-weighted model and should not be interpreted as risk ratios.

**Table 3:** Primary survey-weighted multivariable model for in-hospital mortality.

Effect	aOR	95% CI	*p*
Phenotype: ECMO support vs. reference	60.116	33.291–108.558	<0.0001
Phenotype: postoperative MV > 96 h vs. reference	5.284	2.999–9.309	<0.0001
Phenotype: postoperative MV 24–96 h/intubation vs. reference	8.758	4.444–17.261	<0.0001
Phenotype: other invasive support vs. reference	4.847	2.949–7.965	<0.0001
Multisystem involvement: yes vs. no	1.393	1.070–1.815	0.0139
Age: neonate vs. 1–4 y	3.165	1.799–5.566	<0.0001
Age: infant vs. 1–4 y	1.631	0.863–3.083	0.1316
Age: 5–9 y vs. 1–4 y	1.222	0.551–2.711	0.6219
Age: 10–14 y vs. 1–4 y	2.590	1.255–5.343	0.0100
Age: 15–17 y vs. 1–4 y	2.167	1.121–4.190	0.0214
Female vs. male	1.098	0.799–1.510	0.5650
Comorbidity: 1–2 vs. 0	1.875	1.349–2.606	0.0002
Comorbidity: ≥3 vs. 0	3.134	1.994–4.926	<0.0001
Procedure: neurologic/ENT vs. cardiovascular	2.343	1.428–3.845	0.0008
Procedure: musculoskeletal vs. cardiovascular	0.605	0.255–1.435	0.2544
Procedure: GI/hepatobiliary vs. cardiovascular	1.739	1.060–2.853	0.0284
Procedure: respiratory vs. cardiovascular	1.672	1.031–2.711	0.0372
Procedure: others vs. cardiovascular	1.278	0.848–1.925	0.2409

Note: Type III design-based *p*-values for the overall effects were phenotype < 0.0001, multisystem 0.0139, age < 0.0001, sex 0.5650, comorbidity < 0.0001, and procedure domain of 0.0060. Full model diagnostics are retained in Appendix A for reproducibility but are not interpreted as predictive performance.

**Table 4:** Prespecified sensitivity analyses for respiratory-support phenotypes and multisystem involvement.

Model	*n*/Deaths	ECMO aOR	>96 h aOR	24–96 h/Intub aOR	Other Support aOR	Multisystem aOR
Primary adjusted	6334/216	60.12 (33.29–108.56)	5.28 (3.00–9.31)	8.76 (4.44–17.26)	4.85 (2.95–7.97)	1.39 (1.07–1.82)
Without procedure domain	6334/216	59.00 (34.89–99.76)	5.75 (3.24–10.19)	9.09 (4.70–17.58)	5.29 (3.25–8.61)	1.27 (0.98–1.65)
Post-major-OR ECMO timing	6283/200	60.74 (32.77–112.58)	5.22 (2.96–9.22)	8.65 (4.38–17.07)	4.81 (2.92–7.92)	1.45 (1.12–1.88)
Alternative hierarchy: MV > 96 h before ECMO	6334/216	51.72 (27.42–97.56)	7.99 (4.74–13.48)	8.55 (4.36–16.76)	4.74 (2.87–7.83)	1.46 (1.12–1.89)
Alternative hierarchy: 24–96 h/intubation before >96 h	6334/216	60.46 (33.46–109.26)	4.20 (2.28–7.75)	8.82 (4.81–16.17)	4.86 (2.96–7.98)	1.39 (1.07–1.80)
Last-major-OR timing anchor	6334/216	59.49 (32.95–107.40)	6.25 (3.48–11.21)	9.26 (4.15–20.65)	4.87 (2.96–8.02)	1.41 (1.08–1.83)
No-comorbidity, all eligible	6354/217	65.55 (36.47–117.81)	5.52 (3.11–9.83)	8.81 (4.43–17.49)	5.01 (3.05–8.24)	1.44 (1.11–1.88)
No-comorbidity, comorbidity-complete	6334/216	65.01 (36.14–116.93)	5.56 (3.12–9.89)	8.82 (4.44–17.51)	5.05 (3.07–8.30)	1.46 (1.12–1.90)

Note: Values are adjusted odds ratios with 95% confidence intervals. All models use the KID complex survey design. The alternative hierarchy and last-major-OR models use the same final 6334-record cohort as the primary model. The final two rows omit comorbidity by design to assess selection from missing linkage.

## Data Availability

The data analyzed in this study are available for purchase from the Agency for Healthcare Research and Quality (AHRQ)’s Healthcare Cost and Utilization Project (HCUP) at https://hcup-us.ahrq.gov/kidoverview.jsp (accessed on 12 December 2025). The authors do not have permission to redistribute the underlying dataset. Analytic code is available from the corresponding authors on reasonable request.

## Abbreviations

AHRQ	Agency for Healthcare Research and Quality
CCSR	Clinical Classifications Software Refined
CI	Confidence interval
ECMO	Extracorporeal membrane oxygenation
HCUP	Healthcare Cost and Utilization Project
KID	Kids’ Inpatient Database
MODS	Multiple organ dysfunction syndrome
MV	Mechanical ventilation
OR	Operating room/odds ratio, according to context
PDI-09	Pediatric Quality Indicator 09
PRISM	Pediatric Risk of Mortality
PODIUM	Pediatric Organ Dysfunction Information Update Mandate
pSOFA	Pediatric Sequential Organ Failure Assessment

## Appendix A

This appendix is part of the manuscript and documents the frozen cohort definitions, timing audit, AHRQ comorbidity provenance, complete model coefficients, sensitivity analyses, secondary course characteristics, cohort-selection assessment, phenotype overlap, operative-anchor robustness, survey-design checks, and SAS 9.4 reproducibility details.

### Appendix A.1. Cohort and Respiratory-Support Definitions

**Table A1:** Frozen cohort and primary phenotype operational definitions.

Construct	Code/Source	Operational Rule
Cohort diagnosis	CCSR RSP017	Postprocedural or postoperative respiratory system complication
Operative restriction	PCLASS 3 or 4	Major diagnostic or major therapeutic procedure
Major-OR anchor	First dated PCLASS 3/4 procedure day	Primary timing anchor; same-day major procedures share the common day; last-major-OR anchor tested in sensitivity analysis
Primary age range	≤17 years	Aligned with AHRQ PDI-09 pediatric age criterion
Outcome	KID DIED	In-hospital mortality; no old-QI mortality field imported
ECMO phenotype	Hospitalization-level ECMO support	Highest-priority primary phenotype to isolate extracorporeal-support hospitalizations; alternative hierarchy tested
MV > 96 h phenotype	5A1955Z	Last dated occurrence on/after first major OR
MV 24–96 h phenotype	5A1945Z	Last dated occurrence ≥ 2 days after first major OR
Postoperative intubation	0BH13EZ; 0BH18EZ; 0BH17EZ	Last dated occurrence ≥ 1 day after first major OR
Other invasive support	Remaining coded MV/intubation support	Did not meet higher-priority postoperative timing phenotype
Reference phenotype	No coded MV/intubation/ECMO	No coded invasive mechanical ventilation, qualifying intubation, or ECMO

Note: AHRQ tracheostomy creation codes audited separately were 0B110F4, 0B114Z4, 0B110Z4, 0B113F4, 0B113Z4, and 0B114F4. Tracheostomy was not part of the primary phenotype hierarchy because timing and receipt are conditional on survival and prolonged course.

### Appendix A.2. Cohort Derivation and Timing Audit

**Table A2:** Cohort derivation.

Step	*n*	Comment
All KID 2022 discharges	3,009,812	100% sample
RSP017-positive discharges	10,543	Broad postprocedural respiratory complication group
Operative RSP017 cohort	7378	Final operative source cohort
Identifiable first major OR	7179	97.30% of operative cohort
Primary age ≤ 17 cohort	6358	Before comorbidity/mortality completeness
Linked AHRQ comorbidity	6338	99.69% of primary cohort
Final adjusted model	6334	216 deaths

### Appendix A.3. Timing-Anchor Quality Audit

**Table A3:** Timing-anchor quality audit.

Audit Item	Result	Interpretation
Major-OR anchor shift > 0 days	1990/7179 (27.72%)	Earliest inpatient procedure often preceded first major OR
Exact MV > 96 h code present (5A1955Z)	1908	1064 met postoperative timing; 800 did not; 44 were timing-unclassifiable
Corrected postoperative MV > 96 h	1064	AHRQ timing applied using the first-major-OR anchor
Corrected postoperative MV 24–96 h	126	AHRQ timing applied
Corrected postoperative intubation	475	AHRQ timing applied
MV > 96 h code present but not meeting postoperative timing	800	Secondary hospital-course marker
Timing-unclassifiable operative records	199	No usable major-OR anchor

### Appendix A.4. AHRQ Comorbidity Provenance and Missingness

**Table A4:** Comorbidity linkage and validation.

Item	Result	Interpretation
Primary age-eligible cohort	6358	Current phenotype file + clean KID Core
Matched to deduplicated AHRQ-QI source	6338 (99.685%)	KEY/HOSPID linkage after one-row-per-discharge collapse
Internally inconsistent comorb_NonWt duplicate pairs	0	All duplicate copies agreed on the nonweighted count
PD09_NonWt_1 threshold verification	2997/2997	Perfect 1–2 category concordance in matched cohort
PD09_NonWt_2 threshold verification	601/601	Perfect ≥3 category concordance in matched cohort
Unmatched records	20	1 death, 1 ECMO, 5 multisystem
Final model after age/mortality completeness	6334	216 deaths

Note: The historical AHRQ-QI analytic source supplied only comorb_NonWt and PD09_NonWt category flags to the current analysis. Its DIED and AGEDAY fields were not used because separate audits demonstrated disagreement with the current KID Core. Mortality was sourced exclusively from the current KID DIED variable; neonatal age classification used AGE_NEONATE from the current KID Core.

### Appendix A.5. Cohort Selection and Included-Versus-Excluded Assessment

**Table A5:** Descriptive comparison of final-model included and excluded age-eligible records.

Characteristic	Included	Excluded
Final-model status	6334 included	24 excluded
Female sex	2646 (41.77%)	11 (45.83%)
Neonate, first 28 days	1150 (18.16%)	4 (16.67%)
Infant, 29 d- < 1 y	1448 (22.86%)	5 (20.83%)
Age 1–4 y	1284 (20.27%)	3 (12.50%)
Age 5–9 y	798 (12.60%)	3 (12.50%)
Age 10–14 y	872 (13.77%)	4 (16.67%)
Age 15–17 y	782 (12.35%)	3 (12.50%)
AGE = 0, neonatal status unresolved	0	2 (8.33%)
ECMO support	205 (3.24%)	2 (8.33%)
Postoperative MV > 96 h	846 (13.36%)	3 (12.50%)
Postoperative MV 24–96 h/intubation	227 (3.58%)	0
Other invasive support	1433 (22.62%)	12 (50.00%)
No coded MV/intubation/ECMO	3623 (57.20%)	7 (29.17%)
Multisystem involvement	1313 (20.73%)	7 (29.17%)
Cardiovascular procedure domain	2352 (37.13%)	6 (25.00%)
Neurologic/ENT domain	757 (11.95%)	4 (16.67%)
Musculoskeletal domain	616 (9.73%)	1 (4.17%)
GI/hepatobiliary domain	557 (8.79%)	2 (8.33%)
Respiratory procedure domain	966 (15.25%)	5 (20.83%)
Other principal procedure domain	1086 (17.15%)	6 (25.00%)
Observed in-hospital death	216/6334 (3.41%)	1/22 with known mortality (4.55%)
Length of stay, median (IQR)	12 d (4–37); *n* = 6279	38 d (13–91); *n* = 23

Note: Exclusions were mutually exclusive: 20 records lacked linked AHRQ nonweighted comorbidity information, 2 had missing mortality, and 2 had unresolved AGE_NEONATE status. No significance tests were performed because the excluded group contained only 24 records.

### Appendix A.6. Phenotype Overlap and Alternative Definitions

**Table A6:** Candidate phenotype overlap before hierarchical assignment.

Pre-Hierarchy Overlap Pattern	*N*	Primary Hierarchical Assignment
MV > 96 h + MV 24–96 h/intubation	215	Assigned to postoperative MV > 96 h
ECMO + MV > 96 h	56	Assigned to ECMO
ECMO + other invasive support	45	Assigned to ECMO
ECMO + MV > 96 h + MV 24–96 h/intubation	22	Assigned to ECMO
ECMO + MV 24–96 h/intubation	12	Assigned to ECMO
Total meeting ≥ 2 candidate categories	350 (5.50% of 6358)	328 met two categories; 22 met three

The hierarchy was used only to create mutually exclusive analytic categories and was not intended as an ordinal severity scale. Alternative hierarchy models are summarized in Table A8.

### Appendix A.7. Full Primary Model

**Table A7:** Complete primary survey-weighted logistic regression estimates.

Effect	Beta	SE	aOR	95% CI	*p*
Phenotype: ECMO support vs. reference	4.0963	0.3014	60.116	33.291–108.558	<0.0001
Phenotype: postoperative MV > 96 h vs. reference	1.6646	0.2889	5.284	2.999–9.309	<0.0001
Phenotype: postoperative MV 24–96 h/intubation vs. reference	2.1700	0.3461	8.758	4.444–17.261	<0.0001
Phenotype: other invasive support vs. reference	1.5783	0.2534	4.847	2.949–7.965	<0.0001
Multisystem involvement: yes vs. no	0.3317	0.1348	1.393	1.070–1.815	0.0139
Age: neonate vs. 1–4 y	1.1521	0.2880	3.165	1.799–5.566	<0.0001
Age: infant vs. 1–4 y	0.4895	0.3245	1.631	0.863–3.083	0.1316
Age: 5–9 y vs. 1–4 y	0.2004	0.4064	1.222	0.551–2.711	0.6219
Age: 10–14 y vs. 1–4 y	0.9516	0.3694	2.590	1.255–5.343	0.0100
Age: 15–17 y vs. 1–4 y	0.7736	0.3362	2.167	1.121–4.190	0.0214
Female vs. male	0.0935	0.1625	1.098	0.799–1.510	0.5650
Comorbidity: 1–2 vs. 0	0.6287	0.1678	1.875	1.349–2.606	0.0002
Comorbidity: ≥3 vs. 0	1.1422	0.2307	3.134	1.994–4.926	<0.0001
Procedure: neurologic/ENT vs. cardiovascular	0.8516	0.2526	2.343	1.428–3.845	0.0008
Procedure: musculoskeletal vs. cardiovascular	−0.5020	0.4404	0.605	0.255–1.435	0.2544
Procedure: GI/hepatobiliary vs. cardiovascular	0.5536	0.2524	1.739	1.060–2.853	0.0284
Procedure: respiratory vs. cardiovascular	0.5139	0.2466	1.672	1.031–2.711	0.0372
Procedure: others vs. cardiovascular	0.2451	0.2089	1.278	0.848–1.925	0.2409

Note: Model information: 3,009,812 observations read; 6334 used; 95 strata; 3811 clusters; weight = DISCWT; variance estimation = Taylor series; NOMCAR; Fisher scoring; convergence criterion GCONV = 1 × 10^−8^ satisfied. AIC = 2017.013; SC = 2151.202; −2 log likelihood = 1979.013; c = 0.845.

### Appendix A.8. Sensitivity Analyses

**Table A8:** Sensitivity-analysis summary.

Model	*n*/Deaths	ECMO	>96 h	24–96 h/Intub	Other	Multisystem
Primary adjusted	6334/216	60.12 (33.29–108.56)	5.28 (3.00–9.31)	8.76 (4.44–17.26)	4.85 (2.95–7.97)	1.39 (1.07–1.82)
Without procedure domain	6334/216	59.00 (34.89–99.76)	5.75 (3.24–10.19)	9.09 (4.70–17.58)	5.29 (3.25–8.61)	1.27 (0.98–1.65)
Post-major-OR ECMO timing	6283/200	60.74 (32.77–112.58)	5.22 (2.96–9.22)	8.65 (4.38–17.07)	4.81 (2.92–7.92)	1.45 (1.12–1.88)
Alternative hierarchy: MV > 96 h before ECMO	6334/216	51.72 (27.42–97.56)	7.99 (4.74–13.48)	8.55 (4.36–16.76)	4.74 (2.87–7.83)	1.46 (1.12–1.89)
Alternative hierarchy: 24–96 h/intubation before >96 h	6334/216	60.46 (33.46–109.26)	4.20 (2.28–7.75)	8.82 (4.81–16.17)	4.86 (2.96–7.98)	1.39 (1.07–1.80)
Last-major-OR timing anchor	6334/216	59.49 (32.95–107.40)	6.25 (3.48–11.21)	9.26 (4.15–20.65)	4.87 (2.96–8.02)	1.41 (1.08–1.83)
No-comorbidity, all eligible	6354/217	65.55 (36.47–117.81)	5.52 (3.11–9.83)	8.81 (4.43–17.49)	5.01 (3.05–8.24)	1.44 (1.11–1.88)
No-comorbidity, comorbidity-complete	6334/216	65.01 (36.14–116.93)	5.56 (3.12–9.89)	8.82 (4.44–17.51)	5.05 (3.07–8.30)	1.46 (1.12–1.90)

Note: The alternative hierarchy and last-major-OR analyses use the same final 6334-record cohort as the primary model. The final two rows directly assess selection from records without linked AHRQ comorbidity information. All estimates are survey-weighted.

### Appendix A.9. Six-Level Component Sensitivity

**Table A9:** Six-level sensitivity separating postoperative MV 24–96 h from qualifying postoperative intubation.

Category/Effect	Adjusted OR	95% CI
ECMO support	60.90	33.60–110.38
Postoperative MV > 96 h	5.30	3.01–9.35
Postoperative MV 24–96 h	2.38	0.61–9.34
Qualifying postoperative intubation	13.76	6.92–27.36
Other invasive support	4.87	2.96–8.01
Multisystem involvement	1.41	1.08–1.84

Note: The six-level sensitivity used the same final 6334-record cohort and the same adjustment set as the primary model. The 24–96 h ventilation estimate was imprecise, whereas qualifying postoperative intubation retained a strong association with mortality.

### Appendix A.10. Operative-Anchor and Survey-Design Audit

**Table A10:** Operative-anchor and survey-design robustness checks.

Audit Item	Result	Interpretation
One dated major procedure, final cohort	2115/6334 (33.39%)	Primary first-major-OR anchor unambiguous
Multiple major procedures, same day	2619/6334 (41.35%)	Shared hospital day used; within-day order unavailable
Major procedures spanning >1 day	1600/6334 (25.26%)	First dated major OR primary; last major OR tested in sensitivity
Last-minus-first major OR interval, primary age cohort	Median 0 d; Q3 1 d; P90 24 d	Substantial right tail prompted alternate-anchor analysis
Reclassified using last-major-OR anchor	502/6334 (7.93%)	Adjusted phenotype associations remained similar
Full KID survey variables missing	DISCWT = 0; KID_STRATUM = 0; HOSP_KID = 0	No missing design information
Full KID design	95 strata; 3811 hospital clusters	Minimum 2 sampled hospitals per full-KID stratum
Final analytic cohort represented	51 strata; 400 hospitals	Full 95-stratum/3811-cluster design retained for variance estimation

Note: Survey models used Taylor-series variance estimation with STRATA KID_STRATUM, CLUSTER HOSP_KID, WEIGHT DISCWT, and NOMCAR. The response was set missing outside the analytic subpopulation rather than physically subsetting the design file.

### Appendix A.11. Secondary Analyses and Reproducibility Notes

**Table A11:** Secondary hospital-course and timing analyses.

Subgroup	*n*	Deaths	Mortality	Interpretation
Phenotype 4: no non-postop >96 h code	866	10	1.15%	Reference subgroup
Phenotype 4: non-postop >96 h code	567	49	8.64%	*p* < 0.0001 vs. no non-postop >96 h code
Postoperative tracheostomy absent	6098	198	3.25%	Descriptive only
Postoperative tracheostomy present	258	19	7.36%	Descriptive; survivor/time bias
ECMO before major OR	46	14	30.43%	Timing sensitivity excluded
ECMO on/after major OR	155 with known DIED	58	37.42%	Primary ECMO hospital-course subgroup
ECMO timing unknown	5	2	40.0%	Timing sensitivity excluded

Note: Denominators differ by row. Phenotype 4 subgroups are drawn from the final adjusted model (*n* = 6334); tracheostomy and ECMO timing subgroups are drawn from the age-eligible cohort with known mortality. These analyses are descriptive and should not be interpreted causally.

*Reproducibility Notes*
  - Software: SAS 9.4 with SAS/STAT. Primary modeling used PROC SURVEYLOGISTIC.
  - Survey design: STRATA KID_STRATUM; CLUSTER HOSP_KID; WEIGHT DISCWT; NOMCAR.
  - Subpopulation variance: the full KID design file was retained; the response was set missing outside the analytic subpopulation rather than physically subsetting the design file.
  - No outcome-driven phenotype regrouping was performed. The primary phenotype hierarchy was frozen before the mortality models; alternative hierarchy analyses were performed only as sensitivity analyses.
  - The primary model included phenotype + multisystem + age_hcup + sex + comorb3 + proc_domain. The prespecified sensitivity model omitted proc_domain.
  - All *n* values in manuscript tables are unweighted sampled discharges unless explicitly identified as survey-weighted. Odds ratios and inferential statistics use the KID complex survey design. Survey-weighted absolute mortality and absolute mortality differences are labeled explicitly.
  - The current study does not reproduce PDI-09. PDI-09 specifications were used only to anchor postoperative respiratory-support timing within the broader operative RSP017 cohort.
